# Evaluating combined effects of pesticide and crop nutrition (with N, P, K and Si) on weevil damage in East African Highland Bananas

**DOI:** 10.1371/journal.pone.0282493

**Published:** 2023-03-10

**Authors:** Hannington Bukomeko, Godfrey Taulya, Antonius G. T. Schut, Gerrie W. J. van de Ven, Jerome Kubiriba, Ken Giller

**Affiliations:** 1 Plant Production Systems Group, Wageningen University & Research, Wageningen, The Netherlands; 2 International Institute of Tropical Agriculture, Kampala, Uganda; 3 National Agricultural Research Laboratories, Kawanda, Uganda; Manonmaniam Sundaranar University, INDIA

## Abstract

Banana weevil (*Cosmopolites sordidus*, Germar) is a major pest in East African Highland Banana. The influence of crop nutritional status on weevil damage is poorly understood. Nutrient availability affects the nutritional quality of plants for weevils and may affect weevil damage. Here, we evaluate the effect of insecticides alone and in combination with fertilisers (N, P, K and Si) on weevil damage using data from two experiments in central and southwest Uganda. In the first experiment, we varied chlorpyrifos and application rates of N, P and K. In the second experiment, we varied the application rates of K and Si. Treatment effects were analysed using generalised linear mixed models with a negative binomial distribution. In the first experiment, chlorpyrifos reduced and N increased weevil damage, while P and K had no significant effect. In the K or Si application rates reduced weevil damage compared with the control. We conclude that the combined application of chlorpyrifos with K and Si fertilisers can contribute to weevil damage control on sites with low nutrient availability and should form part of integrated weevil management in bananas. Future studies should assess how much reduction in insecticide use is possible in EAHB with judicious input rates.

## 1. Introduction

The productivity of East African Highland Bananas (EAHBs) in Uganda is 10 to 20 t ha^-1^ y^-1^ [1], barely a third of the attainable yield of 60–70 t ha^-1^ y^-1^ [2]. Yield is mostly constrained by drought, nutrient limitations and pest damage [1]. The banana weevil (*Cosmopolites sordidus*, Germar) is a major banana pest that can cause up to 44% yield loss by the third crop cycle [3]. Weevil larvae damage the corm and, hence, interfere with nutrient uptake and transport, worsening nutrient shortages [4]. Sometimes, EAHBs may not even respond to fertilizers without controlling weevil damage first [5].

Weevil damage control options include chemical control, cultural control practices (e.g. crop sanitation and clean planting materials) and other agronomic practices like good nutritional management [4]. None of these methods is completely effective, hence the advice for integrated pest management (IPM) with a mix of crop management actions that complement each other to augment weevil damage control [4]. IPM reduces the need for insecticides, the risk of insecticide resistance and can limit unintended negative effects on non-target species. Using a combination of fertilisers and insecticides, Kagoda et al. [6] attempted to rehabilitate a heavily weevil-infested plantation but failed because the weevil control interventions started too late (beyond the 5^th^ crop cycle) and instead recommended replanting rather than rehabilitating. It, therefore, remains to be seen if the combined application of insecticide and fertiliser can contribute to weevil control.

Fertilizer applications and water management affect pest damage by altering the nutritional quality of plants to pests. For example, drought stress enhances pest survival among boring insects but deters free-living chewing insects [7]. High nitrogen (N) intake can promote pest damage by increasing the concentration of primary metabolites, such as amino acids which is a nutritional resource for insects. It makes the plant more palatable, nutritious, and digestible [8]. Conversely, silicon (Si) can suppress damage physically by fortifying cell walls or biochemically by inducing resistance [9,10]. Similarly, potassium (K) can reduce insect damage because of its role in metabolic pathways, some of which upregulate defence mechanisms or promote the synthesis of secondary metabolites that make plants less palatable to insect pests [11].

In EAHB, previous studies on weevils and nutrition showed that NPK fertilizer use does not improve productivity in weevil-infested plants [5] nor affect weevil damage [12]. The weevils attacked vigorously growing plants just as much as drought and nutrient-stressed plants [13]. These studies, however, applied low rates of fertilizers and combined nutrient rates in a way that masks individual nutrient effects. For example, [12] combined equal amounts of N and K at a rate of 50 kg ha^-1^ y^-1^. This rate is low and lacks variation in rates of individual nutrients, making it impossible to segregate N and K effects. We are also yet to understand the effects of water or Si on weevil damage. Si alleviates other biotic stresses in bananas like Xanthomonas wilt disease [14] in EAHBs caused by *Xanthomonas campestri*s pv. *musacearum*, Fusarium wilt disease [15] caused by *Fusarium oxysporum* and, Black sigatoka [16] in Grand Nain bananas caused by *Mycosphaerella fijiensis* (Morelet). This study aimed to evaluate the effect of the most used insecticide chlorpyrifos in combination with water, N, K and Si on weevil damage in EAHBs. This knowledge can inform best practices for integrated weevil management.

## 2. Materials and methods

### 2.1. Study sites

The first field trial (referred to below as the Nutrient Omission Trial) was established on land without a history of EAHB cropping in two study areas: Ntungamo (0°54′ S, 30°15′ E, 1405 m.a.s.l) in south-western Uganda and Kawanda (0°25′ N, 32°31′ E, 1156 m.a.s.l) in central Uganda. The trial was planted between October and December 2004 and monitored until 2009. A second trial (referred to as the Potassium Response Trial) was established at Kawanda in December 2018 and monitored until September 2021. The soil type in Ntungamo is a Lixic Ferralsol while the soil in Kawanda is a Haplic Ferralsol. The soils were generally of low fertility (Table 1). Rainfall patterns are bimodal with dry spells from June to August and December to February. Rainfall in Ntungamo ranges from 935 to 1380 mm while rainfall in Kawanda ranged from 1034 to 1663 mm [17]. The climate is typical for much of the EAHB growing areas in the mid-altitude East African highlands with a mean daily minimum and maximum temperature that ranges from 13 to 17°C and 26 to 27°C, respectively [18,19].

**Table 1:** Chemical properties of the top 30 cm of soils in the experimental sites.

Soil Chemical properties	Location					
	Kawanda^§^		Ntungamo^§^		Kawanda^†^	
	Range (Mean)	Class	Range (Mean)	Class	Range (Mean)	Class
pH (1:2.5)	4.9–6.2 (5.5)	Strongly acidic	4.6–5.6 (4.8)	Strongly acidic	5.3–6.3 (5.8)	Moderately acidic
Organic matter (%)	1.0–4.6 (2.6)	Medium	0.14–1.9 (0.7)	Very Low	0.82–4.7 (2.19)	Medium
Nitrogen (%)	0.005–0.2 (0.1)	Low	0.04–0.14 (0.07)	Low	0.077–0.20 (0.11)	Low
Extractable P (mg kg^-1^)	0.7–8.6 (1.8)	Low	0.61–38.0 (3.52)	Very Low	<0.05	Very Low
Exchangeable K (cmol_c_ kg^-1^)	0.04–1.0 (0.4)	Medium	0.02–0.36 (0.12)	Low	0.054–0.351 (0.19)	Low
Exchangeable Ca (cmol_c_ kg^-1^)	2.2–8.6 (4.5)	Low	0.47–7.4 (1.7)	Low	2.08–5.462 (3.6)	Low
Exchangeable Mg (cmol_c_ kg^-1^)	0.9–2.9 (1.48)	Medium	0.01–1.6 (0.45)	Low	0.897–1.893 (1.34)	Medium

^§^ Nutrition Omission Trial.

^†^ Potassium Response Trial.

### 2.2 Experimental designs and data collection

#### 2.2.1. Nutrient omission trial (2004–2009)

A randomized complete block design was used with four blocks across the slope. Each block had 10 treatments (Table 2) and each treatment consisted of 35 mats laid out in a 5 × 7 arrangement occupying an area of 315 m^2^. The inner 3 × 5 mats were sampled. EAHBs of the variety Kisansa were used–a variety susceptible to weevil damage. The primary nutrients N-P-K-Mg were applied using the mineral fertilizers urea (CH_4_N_2_O), muriate of potash (KCl), triple superphosphate (Ca (H_2_PO_4_)2·H_2_O), and kieserite (MgSO_4_) respectively. Micro-nutrients were applied using sodium molybdate (Na_2_MoO_4_), borax (Na₂ [B₄O₅ (OH) ₄] ·8H₂O) and zinc sulphate (ZnSO_4_). The nutrient rates in this trial were selected to enable QUEFTS modelling and quantify banana yield response to nutrient fertilisers. For treatments 1, 5, 8 and 10 (Table 2) with the highest rates of fertilizer, N and K fertilizers were applied in four splits, two per rainy season. Fertilizers for all other treatments were applied in two splits, one at the start of each rainy season. Weevils were controlled using chlorpyrifos insecticide of the brand Dursban [20]–sprayed at a recommended rate of 1.03 g per mat per month. Micro-bunds (soil heaped up to 30 cm high around the plot area) were installed between plots to prevent runoff/run-on.

**Table 2:** Treatments applied in the nutrient omission trial.

		Treatments								
Application	1	2	3	4	5	6	7	8	9	10
N (kg ha^-1^ y^-1^)	400	-	-	150	400	400	400	400	-	400
P (kg ha^-1^ y^-1^)	50	-	50	50	-	50	50	50	-	50
K (kg ha^-1^ y^-1^)	600	-	600	600	600	-	250	600	-	600
Other nutrients	1	-	1	1	1	1	1	-	-	1
Pesticide	1	1	1	1	1	1	1	1	-	-

Treatments 1–7 were also used in Nyombi [19] and treatments 1–4 and 6–7 were also used in Taulya [17]. Other nutrients included magnesium, zinc, boron and molybdenum.

Weevil damage was assessed on freshly harvested corms of EAHBs [21] for every crop cycle. A banana plant, is composed of an underground stem called a "corm" whose tissues can be distinguished into a central cylinder surrounded by an outer cortex. Out of the corm, multiple aerial shoots, known as pseudo-stems emerge sporadically and together they constitute a banana mat. The reproductive growth phase starts when an inflorescence emerges from the top of the pseudo stem and develops into a bunch, which at maturity (banana fingers start to ripen) culminates in the death and decay of the pseudo stem and the portion of corm attached to it. The cohort of pseudo stems in a given field that emerge from their respective corms within the same season constitutes a banana crop cycle even though these may be harvested at different periods of time since their growth and development rates can be different. Weevil assessment was done on part of the corm directly attached to the harvested pseudo-stem. To assess weevil damage, two cross-sectional cuts were made through the corm at the collar, i.e., at the junction of the pseudo-stem and corm, and 5 cm below the collar. For each cross-section, the percentage area of tissue consumed by weevil larvae in the central cylinder and the cortex were estimated, giving two damage estimates per cross-section. Overall weevil damage was determined as the mean of these four estimates.

Nyombi [19] used data from this nutrient omission trial to describe the biomass growth response to fertilizer inputs, while Taulya [22] used it to study the effect of nutrients on drought tolerance of EAHB. We used data from the nutrient omission trial to examine the additional effect of fertilizers on weevil damage on top of pesticide use. The setup of a nutrient omission trial was however not optimal for assessing the effect of potassium on weevil damage because it lacked sufficient variation in potassium levels with the low/moderate nitrogen rate. For this, we considered the potassium response trial where potassium was varied while keeping a moderate rate of nitrogen.

#### 2.2.2. Potassium response trial (2018–2021)

The potassium response trial was used to examine the contribution of K and Si to weevil damage control. This trial had a similar layout as the nutrient omission trial but with only three blocks and had mixed varieties of EAHBs randomly distributed in the trial–all susceptible to weevil damage. Each block had 16 treatment plots and each treatment plot included 15 mats at the start of the experiment. In each block, eight treatment plots were rain-fed, and the other eight plots were drip-irrigated with a pressure-compensating pump. The irrigation was only done during the dry season and each irrigation event supplied 30 litres of water per mat within five hours. It was not applied frequently enough to fully prevent water limitation. The primary nutrients N, P and K were applied using mineral fertilizers urea (CO (NH_2_)_2_), muriate of potash (KCl) and triple superphosphate (Ca (H_2_PO_4_)2·H_2_O). The rate of nitrogen used in this trial was considered moderate while potassium varied from lowest to maximum plausible for bananas. These rates were selected to test the effect of varying K without the likely masking effect of high N. The N was applied in 4 splits (2 times per rainy season, 25 kg N ha^-1^ per application), adding to a total of 100 kg N ha^-1^ y^-1^. P was applied twice a year at the rate of 25 kg P ha^-1^ at the start of each rainy season, adding to a total of 50 kg P ha^-1^ y^-1^. Varying amounts of K (Table 3) were applied in four splits. Si was provided as Elkem B–a Si fertilizer containing 45% Si in the form of SiO_4_ –at the manufacturer’s recommended rate of 300 kg Si ha^-1^ y^-1^ and applied in two splits. Weevils were controlled with the insecticide chlorpyrifos, sprayed monthly. Weevil damage was assessed similarly to section 3.2.1 following Gold et al. [21] starting from December 2019 to September 2021. The weevil damage assessments were done on four of the 15 mats. These four were chosen randomly but the same four mats were assessed throughout the assessment period.

**Table 3:** Treatments applied in the potassium response trial.

Treatments	Water	Si (kg ha^-1^ y^-1^)	K (kg ha^-1^ y^-1^)
1	Irrigated	0	0
2	Irrigated	300	0
3	Irrigated	0	75
4	Irrigated	300	75
5	Irrigated	0	150
6	Irrigated	300	150
7	Irrigated	0	250
8	Irrigated	0	600
9	Rain-fed	0	0
10	Rain-fed	300	0
11	Rain-fed	0	75
12	Rain-fed	300	75
13	Rain-fed	0	150
14	Rain-fed	300	150
15	Rain-fed	0	250
16	Rain-fed	0	600

### 2.3. Data analysis

We visualized the raw data in both trials using a cumulative distribution function of the proportion of weevil damage in the corm for each treatment. To test the effect of predictors on weevil damage, we fitted generalized linear mixed models (GLMM). In the nutrient omission trial, predictor variables were binary in the case of chlorpyrifos use, application of phosphorus (P) and “other nutrients” (magnesium, zinc, boron, molybdenum). N and K were each applied at three rates. There were four crop cycles. In the potassium response trial, the predictor variables were binary in the case of irrigation and Si. K was applied at five rates. There were three crop cycles. The predictor variables were used as fixed factors in the model. The random variables were mats nested in plots, which in turn were nested in blocks. The GLMM used an unstructured variance-covariance matrix where it estimates each variance and covariance directly from the data without constraints [23]. We fitted the GLMM using a negative binomial distribution with a log-link function instead of the Poisson model which was over-dispersed [24]. The negative binomial has a dispersion parameter that relaxes the strict Poisson assumption that mean equals variance [24]. Model diagnostic tests like tests for over dispersion, zero inflation, outliers and patterns in residuals were performed. These tests indicated that the selected model fitted the data well.

For each trial, we compared various combinations of predictors with and without interactions. Models with the interaction between crop cycle and treatments were not significant and we instead considered models with main effects of crop cycle plus the various combination of treatments. Additionally, we considered models specified with the crop cycle as a fixed predictor or as part of the dispersion model and, models specifying nutrient application rates with more than two rates as either categorical or continuous variables. We selected models with the lowest value of Akaike information criteria (AIC) and when AIC was not different, we choose the simpler model [25]. During comparisons, model parameters were estimated using maximum likelihood with Laplace approximation which gives reliable fit statistics but biased variance parameter estimates. After model selection, the final models (Model 1 for the nutrient omission trial & model 2 the for potassium response trial), were refitted with restricted maximum likelihood with Laplace approximation which gives unbiased variance parameter estimates.


Weevildamage∼N+P+K+Insecticide+Othernutrient+(1|Block/Plot/Mat) (1)

Weevildamage∼Cropcycle+Water+K+Si+(1|Block/Plot/Mat) (2)

In both models, a restricted maximum likelihood was used in combination with a negative binomial distribution (family was set to “nbinom2”). In Model 1, N, K and crop cycle were continuous variables while the other variables were categorical with crop cycle specified as part of the dispersion model allowing the dispersion parameter to vary with the crop cycle [26]. In Model 2, all variables were categorical. We used Tukey’s post hoc test to compare contrasts among K application rates in Model 2.

In the tables, the estimate is either positive to indicate an increase or negative to indicate a decrease in the response variable due to the predictor variable associated with the estimate. We back-transformed the estimates from the natural log scale and calculated percentage change according to Eq 3:

$$Percentage\;change=100\times (e^{estimate}-1)$$ (3)

We performed these analyses in R [27] with packages: “ggplot2” [28] for plotting, “glmmTMB” [26,29] for model fitting, “bblme” [30] for AIC comparisons, “DHARMa” [31] for model diagnostic tests, and “multcomp” [32] for post hoc testing.

## 3. Results

### 3.1. Effect of insecticide and NPK on weevil damage in EAHBs

**Fig 1:**
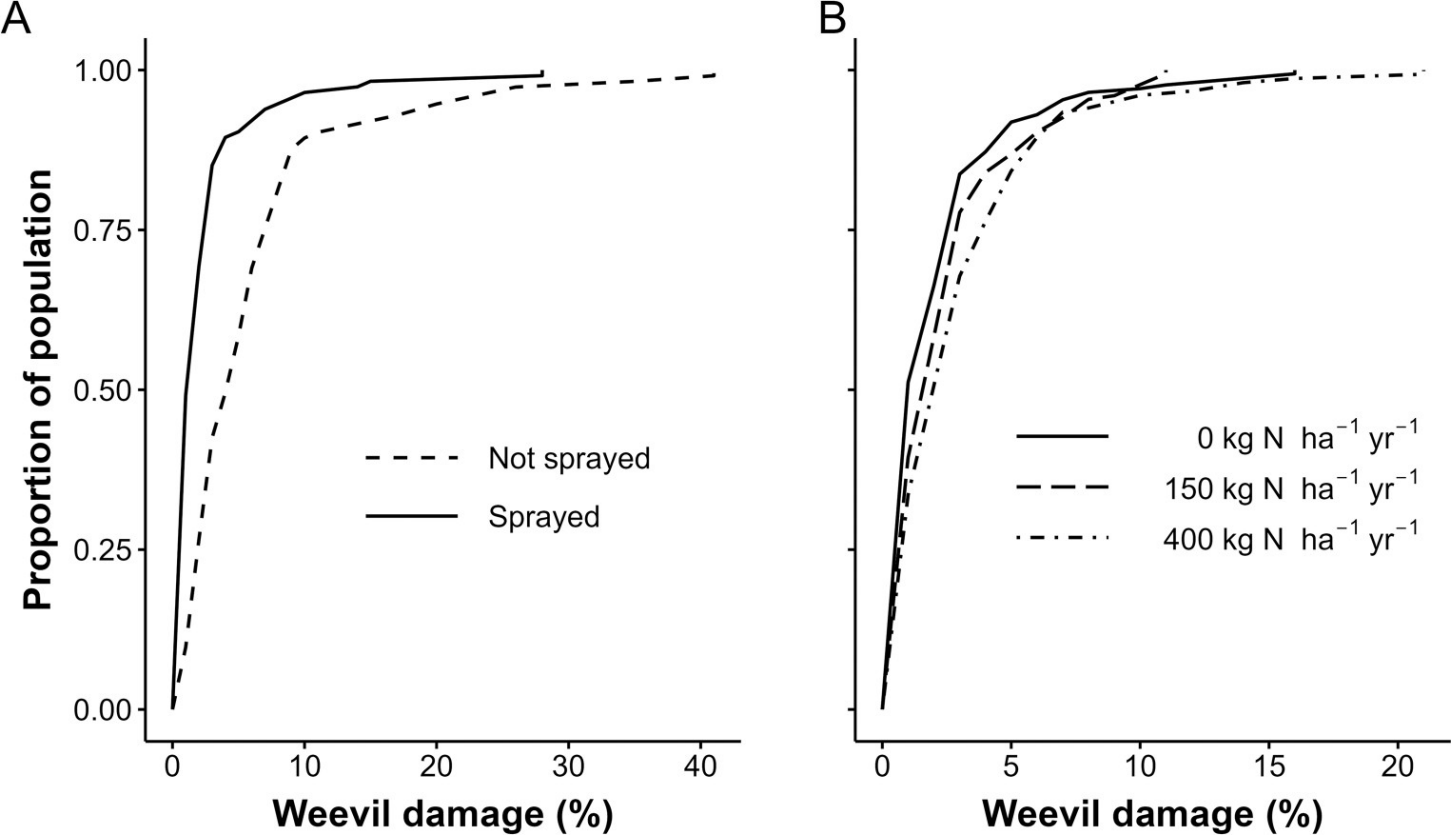
The cumulative distribution function for weevil damage in EAHBs with and without spraying chlorpyrifos (A) and at different N application rates in sprayed treatments (B) in the nutrient omission trial.

In the nutrient omission trial, applying the insecticide chlorpyrifos and N affected weevil damage in EAHBs. For any given level of weevil damage, the proportion of the plant population affected was consistently less in plots sprayed with chlorpyrifos (sprayed but no fertilizer application) than in non-sprayed plots (Fig 1, panel A). This reduction in weevil damage was strongly significant (p = 0.000). The sprayed plants had 57% less damage than plants that were not sprayed (Table 4). The proportion of the plant population sprayed with chlorpyrifos and affected by weevil damage was significantly higher among plants that received 400 kg N ha^-1^ y^-1^; every kg increase in N application per ha per year was associated with a 0.08% increase in weevil damage (Table 4). Applying K, P and “other nutrients” did not significantly affect weevil damage.

**Table 4:** Estimates, standard errors (SE), back-transformed estimates and per cent change in weevil damage as a function of insecticide and fertiliser application to EAHBs in the nutrient omission trial using a GLMM with a negative binomial distribution, log link function and Laplace approximation (n = 1370).

Term	Natural log scale	Back-transformed estimate	% Change	P value
Conditional model				
Fixed effects	Estimate ± SE			
Intercept	1.4775 ± 0.14142	4.3819		0.000
Insecticide	-0.8553 ± 0.0987	0.42512	- 57	0.000
N (kg ha^-1^ y^-1^)	0.0008 ± 0.0003	1.0008	0.08	0.003
P50 (kg ha^-1^ y^-1^)	-0.1262 ± 0.1096	0.8815		0.250
K (kg ha^-1^ y^-1^)	0.0001 ± 0.0002	1.000		0.688
Other nutrients (kg ha^-1^ y^-1^)	-0.0747 ± 0.1405	0.9280		0.595
Dispersion model				
Intercept	-1.4879 ± 0.1886	0.2258		0.000
Crop cycle	0.6225 ± 0.0870	1.8637		0.000
Random effects	standard deviation			
Mat: Plot: Block	0.3261			
Plot: Block	0.0687			
Block	0.1740			

The reference category was zero for the categorical variables insecticide application, P, Other nutrients applied. Nitrogen and Potassium were treated as continuous variables.

### 3.2. Effect of Si, K and irrigation on weevil damage in EAHBs

**Fig 2:**
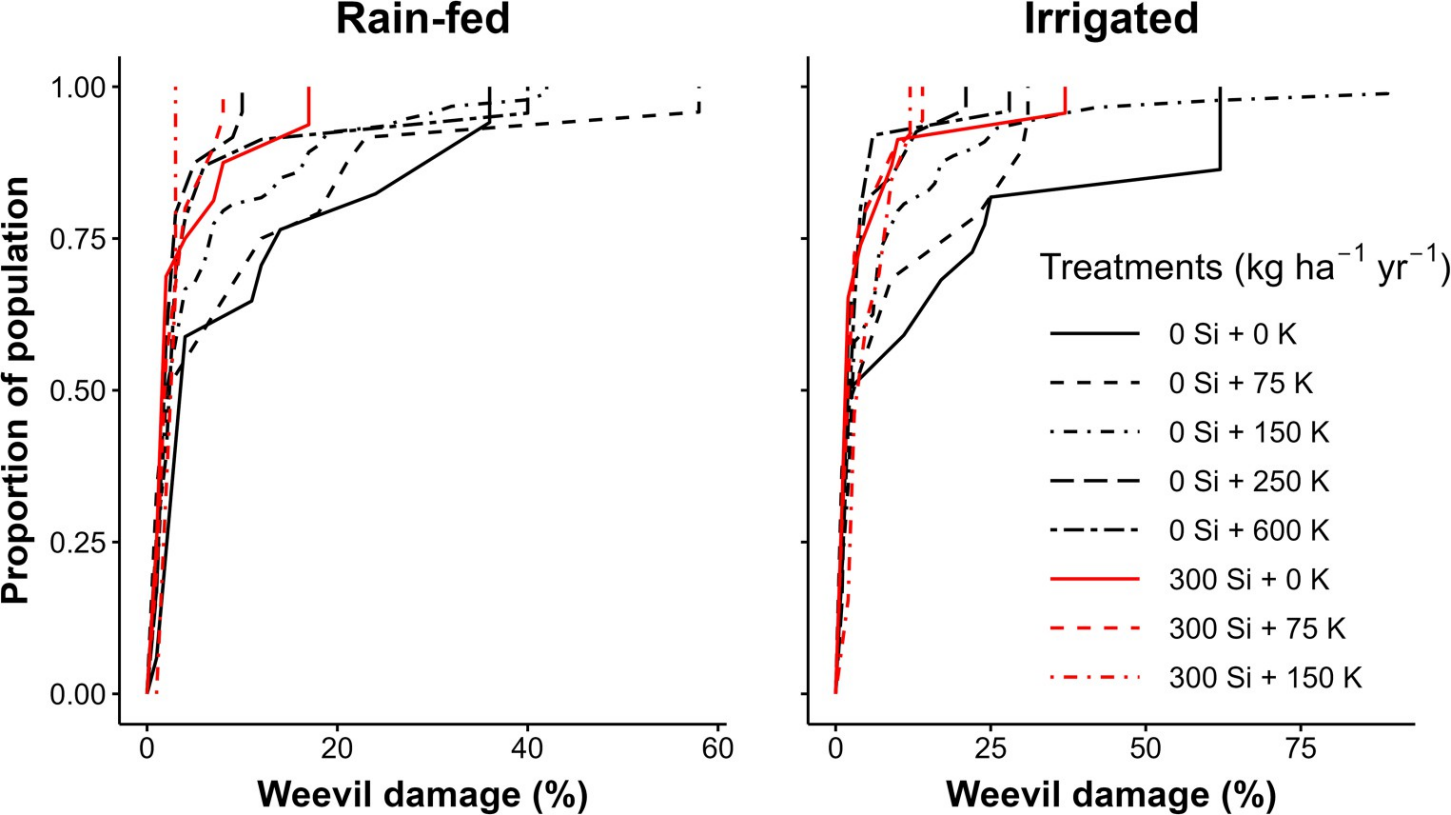
The cumulative distribution function of weevil damage in EAHBs under different water and nutrient treatments. All treatments were sprayed with chlorpyrifos.

In the potassium response trial, higher rates of Si and K were associated with lower weevil damage among plants sprayed with chlorpyrifos (Fig 2). Applying 300 kg Si ha^-1^ y^-1^ was associated with a 45% decrease in weevil damage. Among plants that did not receive Si, the proportion of the plant population affected by weevil damage was generally smaller among plants treated with high K rates such as 250 and 600 kg ha^-1^ y^-1^ than those that received less K. This difference in weevil damage was significant (p < 0.01). When compared to 0 kg K ha^-1^ y^-1^, 250 kg K ha^-1^ y^-1^ was associated with a 67% decrease in weevil damage and 600 kg K ha^-1^ y^-1^ was associated with a 57% decrease in weevil damage (Table 5). These high rates (250 and 600 kg ha^-1^ y^-1^) did not differ significantly from each other (p > 0.05). The effect of irrigation was not significant (Table 5).

**Table 5:** Estimates, standard errors (SE), back-transformed estimates and per cent change in weevil damage as a function of pesticide application combined with irrigation or K or Si fertilizer in the potassium response trial analysed using a GLMM with a negative binomial distribution, log link function and Laplace approximation (n = 449). Pesticide and 100 kg N ha y^-1^ were blankets applied to all treatments shown here.

	Natural log scale	Back-transformed estimate	% Change	P value
Fixed effects	Estimate ± SE			
Intercept	2.1928 ± 0.2775	8.9599		0.000
Crop cycle 2	-1.2333 ± 0.1712	0.2913		0.000
Crop cycle 3	0.2309 ± 0.1821	1.2598		0.205
Irrigated	0.0436 ± 0.1432	1.0445		0.761
Si 300 (kg ha^-1^ y^-1^)	-0.6057± 0.1983	0.5457	- 45	0.002
K 75 (kg ha^-1^ y^-1^)	-0.3795 ± 0.2366	0.6842		0.109
K 150 (kg ha^-1^ y^-1^)	-0.4196 ± 0.2227	0.6573		0.059
K 250 (kg ha^-1^ y^-1^)	-0.9609± 0.2921	0.3825	- 67	0.001
K 600 (kg ha^-1^ y^-1^)	-0.8363± 0.2960	0.4333	- 57	0.005
Random effects	standard deviation			
Mat: Plot: Block (intercept)	0.64274			
Plot: Block (intercept)	0.06756			
Block (intercept)	0.00005			

Dispersion parameter = 0.76.

The reference category is “Crop cycle 1” for Crop cycle, Rain fed for Irrigated and zero for Si and K application rates.

## 4. Discussion

The insecticide chlorpyrifos significantly reduced weevil damage in EAHBs as expected [20]. Chlorpyrifos is a contact insecticide that inhibits nervous-system messaging leading to a nervous-system breakdown that kills the pest. It is, however, not 100% effective because weevils spend a significant time of their life cycle protected inside the banana plant. In the nutrient omission trial, pesticides alone reduced weevil damage by 57%. This study, therefore, combined chemical control with fertiliser use.

Our data show that weevil damage was reduced by 67% with a large rate of K in the potassium response trial where K was combined with moderate rates of N. When high application rates of K were combined with high rates of N–in the nutrient omission trial–the effect of K was not significant. This suggests that the observed effect of K is counteracted by the availability of N, which could explain why previous work [12] did not find a significant effect of NPK on weevil damage in EAHBs when the same amount of K and N were applied. Ssali et al. [12] applied a much lower rate of K (50 kg ha^-1^ y^-1^) compared with that applied in our experiments (up to 600 kg K ha^-1^ y^-1^). Lower rates of K application did not significantly reduce weevil damage in our experiment either. The effect of high rates of K on weevil damage in sites that have low K (e.g. in the potassium response trial) is likely because K enhances the assimilation of carbohydrates into structural material, reducing excess sugars and free proteins in cells hence making them less palatable to weevil larvae. K also facilitates the production of secondary metabolites like phenolic compounds [33] which have been shown to deter weevil-larvae feeding in the resistant dessert banana variety Yagambi-Km5. K deficiency is one of the main production constraints in EAHB in Uganda [1].

In the potassium response trial, we found that plants fertilized with Si had less weevil damage than plants without Si, concurring with findings for other plant-pest interactions [34]. A stronger mechanical barrier [35] and induced resistance [10] may explain the role of Si although Coskun et al. [36] argue that the apoplastic obstruction hypothesis is more likely. The premise is that insects release effectors–insect proteins released into the plant to aid insect attack–into the apoplast [37] where effectors manipulate plant defences [38] and the plant fails to mobilize relevant defence [37,39]. For example, oral secretions of Colorado potato beetle larvae contained bacteria that served as a microbial decoy. The decoy induced the salicylic acid (SA) signalling pathway and, through cross-talk, suppressed Jasmonic acid (JA) mediated defences, which enhanced larval growth [38]. Si, taken up as silicic acid (Si(OH)_4_) and present in the apoplast, obstructs effectors from reaching their targets such that they do not compromise plant defence [36].

In EAHB, Bakaze et al. [40] showed that when weevil larvae fed on resistant varieties, they triggered greater production of phenolics and, greater deposition of lignin and suberin around the damaged area. This response was lacking in the susceptible EAHB variety Mbwazirume until it was artificially supplied with methyl Jasmonate. Following the logic of the apoplastic obstruction hypothesis [36], pest effectors can successfully block the susceptible plants from activating methyl Jasmonate pathways for defence but fail in the resistant variety. Applying Si to susceptible EAHBs may obstruct pest effectors from their targets and allow otherwise susceptible EAHBs, to activate the methyl Jasmonate pathway for defence. To confirm this hypothesis, more experiments are needed that explore the biochemical responses of EAHBs to weevil damage under different fertilizer regimes.

Weevil damage generally increased with N, similar to N effects on other pests including stem borers in rice [41]. These observations concur with the plant vigour hypothesis that suggests that pests prefer to feed on vigorously growing plants [42]. We found that weevil damage increased with N supply most likely because of the high concentration of soluble N-based compounds and free amino acids associated with a high nitrogen supply. A higher concentration of these compounds leads to more pest damage because they make the plant more nutritious and easier to digest for the pest [8]. The bunch yields of EAHB in our experiment did not respond to N applications [22], although drought and impaired uptake due to root constraints may have played a role [17]. Regardless, large N applications were in excess which may have affected the observed increase in weevil damage. The precise (optimal) N application beyond which these negative effects start is still not known.

Though mineral fertiliser use in EAHB is still sparse, efforts to promote fertilisers are picking up along with efforts to intensify banana production. Caution should be taken not to apply very high rates (e.g., 400 kg ha^-1^ y^-1^) of N as this will likely expose EAHBs to higher weevil damage and risks leaking N into the environment and polluting. It is unclear what the optimal ratio and application rates of N and K should be to maximise production and minimize weevil damage. On the other hand, K fertilisers applied for yield gain will come with the added advantage of reducing weevil damage if applied at high rates. For Si, however, its protective role is documented in many studies and now also in EAHBs against weevils but its contribution to yield is not known. Further studies should quantify whether silicon’s protective role translates into yield gains that can cover the cost of Si fertiliser. Given that our results were based on experiments at one site per research question, conducting similar experiments in other sites would confirm whether our results have broader applicability. Filling these knowledge gaps will move us closer to harnessing silicon’s protective role in EAHB.

## 5. Conclusions

We showed that combining K and Si fertiliser use with insecticide can contribute to weevil damage control. Good nutritional management is therefore a key component of the integrated management of weevils in EAHB which might reduce the need for insecticide application. Further studies should investigate how far insecticide use can be reduced in EAHB given good nutritional management.
